# Observations on the Occipital and Superior Maxillary Bones of the African Cranium

**Published:** 1850-01

**Authors:** John Neill


On leave granted, Dr. John Neill presented an abstract of a paper written for the American Journal of Medical Sciences, entitled "*Observations on the Occipital and Superior Maxillary Bones of the Afri-*

*can Cranium.*" A peculiarity in the condyloid process of the occiput was pointed out, which is not generally noticed in works on Anatomy. It consists in a division of the process into two parts by a ridge or groove; showing a tendency in the basi-occipital bone of the fœtal, or young head, to be permanently retained. This peculiarity occurs oftener in the African than in any other head. In this respect there is an analogy to the lower orders of the vertebrata. The superior maxillary bone of the African head is also defective in a ridge which is continuous with the nasal process, and reaches to the anterior nasal spinal in the Caucasian head. In the African, the lower edge of the anterior nares is flat, and in this respect resembles the fœtal head, and the heads of inferior animals.—*Proceed. Acad. Nat. Sci. of Philadelphia*, 1849.

